# Phylogenomic Analyses and Reclassification of Species within the Genus *Tsukamurella*: Insights to Species Definition in the Post-genomic Era

**DOI:** 10.3389/fmicb.2016.01137

**Published:** 2016-07-21

**Authors:** Jade L. L. Teng, Ying Tang, Yi Huang, Feng-Biao Guo, Wen Wei, Jonathan H. K. Chen, Samson S. Y. Wong, Susanna K. P. Lau, Patrick C. Y. Woo

**Affiliations:** ^1^Department of Microbiology, The University of Hong KongHong Kong, China; ^2^Research Centre of Infection and Immunology, The University of Hong KongHong Kong, China; ^3^State Key Laboratory of Emerging Infectious Diseases, The University of Hong KongHong Kong, China; ^4^Carol Yu Centre for Infection, The University of Hong KongHong Kong, China; ^5^Centre of Bioinformatics, Key Laboratory for NeuroInformation of the Ministry of Education, School of Life Science and Technology, University of Electronic Science and Technology of ChinaChengdu, China; ^6^School of Life Sciences, Chongqing UniversityChongqing, China; ^7^Collaborative Innovation Center for Diagnosis and Treatment of Infectious Diseases, The University of Hong KongHong Kong, China

**Keywords:** phylogenomic, reclassification, *Tsukamurella*, species, genomics

## Abstract

Owing to the highly similar phenotypic profiles, protein spectra and 16S rRNA gene sequences observed between three pairs of *Tsukamurella* species (*Tsukamurella pulmonis*/*Tsukamurella spongiae*, *Tsukamurella tyrosinosolvens*/*Tsukamurella carboxy-divorans*, and *Tsukamurella pseudospumae*/*Tsukamurella sunchonensis*), we hypothesize that and the six *Tsukamurella* species may have been misclassified and that there may only be three *Tsukamurella* species. In this study, we characterized the type strains of these six *Tsukamurella* species by tradition DNA–DNA hybridization (DDH) and “digital DDH” after genome sequencing to determine their exact taxonomic positions. Traditional DDH showed 81.2 ± 0.6% to 99.7 ± 1.0% DNA–DNA relatedness between the two *Tsukamurella* species in each of the three pairs, which was above the threshold for same species designation. “Digital DDH” based on Genome-To-Genome Distance Calculator and Average Nucleotide Identity for the three pairs also showed similarity results in the range of 82.3–92.9 and 98.1–99.1%, respectively, in line with results of traditional DDH. Based on these evidence and according to Rules 23a and 42 of the *Bacteriological Code*, we propose that *T. spongiae* Olson et al. 2007, should be reclassified as a later heterotypic synonym of *T. pulmonis* Yassin et al. 1996, *T. carboxydivorans* Park et al. 2009, as a later heterotypic synonym of *T. tyrosinosolvens* Yassin et al. 1997, and *T. sunchonensis* Seong et al. 2008 as a later heterotypic synonym of *T. pseudospumae* Nam et al. 2004. With the advancement of genome sequencing technologies, classification of bacterial species can be readily achieved by “digital DDH” than traditional DDH.

## Introduction

Traditionally, classification of bacteria is performed on the basis of their phenotypic characteristics, such as the appearance of the bacterium under light microscope after Gram staining, growth requirements, biochemical tests, and chemotaxonomic characteristics. However, the results of individual biochemical tests may vary among different strains of the same bacterial species, leading to variations in their biochemical profiles. Therefore, it is often difficult to determine whether a strain belongs to a new species or if it is just a variant of an existing species. In the 1970s, the discovery of conserved small ribosomal RNA (rRNA) gene sequences has marked the beginning of a new era for the study of evolution and classification of living organisms (Fox et al., 1977; Gupta et al., 1983). These rRNA gene sequences are, in general, highly conserved within living organisms of the same genus and species, but different between organisms of different genera and species. With the subsequent invention of PCR and automated DNA sequencing technology in the 1990s, 16S rRNA gene sequences have been widely used for phylogenetic, bacterial classification and identification. Numerous bacterial genera and species have been re-classified and renamed, and many novel bacterial genera and species have been discovered (Woo et al., 2008). However, careful analysis of 16S rRNA data is needed to avoid misinterpretation of species classification, especially in cases where taxonomic claims are proposed on the basis of the 16S rRNA data alone. For example, two strains that share 96% 16S rRNA gene sequence similarity are determined to be members of different species; but in some cases, two strains that share 98% 16S rRNA gene similarity may or may not be members of the same species (Stackebrandt and Goebel, 1994; Palys et al., 1997). As a result, it is difficult to use a universal cut-off value for classifying all bacteria to the species level. Other data sets should always be used in support of the claims.

In view of these problems, DNA–DNA hybridization (DDH) has remained the gold standard for classification in bacterial taxonomy. It represents a universally applicable technique that offers genome-wide comparisons between any bacteria, and a universal DDH value of 70% is proposed to be the criterion for bacterial species delineation (Wayne, 1988). However, this traditional method is time-consuming and labor-intensive, and it is difficult to compare DDH results between different laboratories objectively. Given these drawbacks, bacterial taxonomists have been actively searching for alternative methods to replace the traditional DDH for bacterial classification and species assignment (Goris et al., 2007). In the era of genomics, massive amounts of high-quality bacterial genome sequences can be easily obtained by high-throughput sequencing approach. As a result, different attempts have been made to replace the traditional wet-lab DDH with *in silico* genome-to-genome comparison. Among the various “digital DDH” methods studied, the Genome-To-Genome Distance Calculator (GGDC) and Average Nucleotide Identity (ANI) calculator were shown to yield good correlation with wet-lab DDH values (Goris et al., 2007; Auch et al., 2010). GGDC operates on the same scale as the wet-lab DDH method and use the value of 70% as a cut-off for species delineation (Auch et al., 2010). ANI, on the other hand, estimates the average nucleotide identity between two genomic datasets, and an ANI value in the range of 94–96% has been suggested as the substitute for a wet-lab DDH value of 70% (Konstantinidis and Tiedje, 2005; Goris et al., 2007; Richter and Rosselló-Móra, 2009).

The genus *Tsukamurella* was first proposed in 1988 by Collins and colleagues using both chemotaxonomic data and 16S rRNA gene sequence analysis (Collins et al., 1988), although taxonomic history of the genus can be dated back to 1941, when the first strain of this group of bacteria was isolated from mycetomes and ovaries of bed bugs (*Cimex lectularius*), and was initially named *Corynebacterium paurometabolum* (Steinhaus, 1941). Members of *Tsukamurella* are Gram-positive, aerobic, catalase-positive, and partially acid-fast, with morphology similar to the related genera of the order Actinomycetales, including *Mycobacterium*, *Nocardia*, *Corynebacterium*, *Gordonia*, and *Rhodococcus*. Chemotaxonomic characteristics, such as fatty acid, mycolic acid, menaquinone, and polar lipid composition, have been shown to be particularly useful to study the relatedness of actinomycete and coryneform systematics (Collins and Jones, 1982; Collins et al., 1982). At the time of writing, there are 13 species to be included in the genus *Tsukamurella* according to the current state of the taxonomy, with only seven species associated with human infections. The most common *Tsukamurella* infections in human are related to the presence of indwelling devices (Larkin et al., 1999; Shaer and Gadegbeku, 2001; Schwartz et al., 2002; Almehmi et al., 2004; Bouza et al., 2009; Liu et al., 2011). The disease spectra of *Tsukamurella* further extends to infections related to ophthalmologic diseases (Woo et al., 2003, 2009), and recently, we have reported the discovery of two novel *Tsukamurella* species, *Tsukamurella hongkongensis* and *Tsukamurella sinensis*, from patients with keratitis and conjunctivitis (Teng et al., 2015). *Tsukamurella* is also found in different environmental sources as well as in animals (Maeda et al., 2010; Jiang et al., 2013). However, as nucleotide differences between the 16S rRNA gene sequences of different *Tsukamurella* species are less than 1% (Woo et al., 2003), defining a *Tsukamurella* strain as a novel species relies heavily on DDH results. However, it is important to note that DDH results may vary depending on the identity of the DNA used and any experimental problems cannot be checked in a different laboratory using the same samples, hence it is possible that *Tsukamurella* strains may be wrongly classified on the basis of DDH data alone.

During the process of characterization of *T. hongkongensis* and *T. sinensis* with other type strains of known *Tsukamurella* species, we observed that some of the known *Tsukamurella* species shared identical 16S rRNA gene sequence and highly similar phenotypic characteristics using the same testing method (Teng et al., 2015). Our results showed that almost identical phenotypic profiles were observed between *Tsukamurella pulmonis* CCUG 35732^T^ (Yassin et al., 1996) and *Tsukamurella spongiae* DSM 44990^T^ (Olson et al., 2007), between *Tsukamurella tyrosinosolvens* CCUG 38499^T^ (Yassin et al., 1997) and *Tsukamurella carboxydivorans* JCM 15482^T^ (Park et al., 2009), and between *Tsukamurella pseudospumae* JCM 13375^T^ (Nam et al., 2004) and *Tsukamurella sunchonensis* JCM 15929^T^ (Seong et al., 2003; Euzéby, 2008). However, phenotypic characteristics of these six *Tsukamurella* species obtained from our study were not comparable to those obtained from the original publications because different culture medium, reading interval and incubation conditions of the phenotypic tests have been applied in different studies (Yassin et al., 1996, 1997; Seong et al., 2003; Nam et al., 2004; Olson et al., 2007; Park et al., 2009). We further characterized these strains by matrix-assisted laser desorption ionization time-of-flight mass spectrometry (MALDI-TOF MS), in which the hierarchical cluster analysis dendrogram also revealed the same clustering patterns among these three pairs of *Tsukamurella* type strains. Therefore, we hypothesized that these six *Tsukamurella* species may have been misclassified and some of them may belong to the same *Tsukamurella* species. In fact, when some of these “novel” *Tsukamurella* species were described, only a marginal DDH result of around 62% was observed between *T. tyrosinosolvens* CCUG 38499^T^ and *T. carboxydivorans* JCM 15482^T^ (Park et al., 2009). In this study, we re-characterized these six *Tsukamurella* species by performing the traditional DDH again. In addition, we sequenced their genomes, which represented the first genomes to be sequenced for *T. pulmonis*, *T. spongiae*, *T. tyrosinosolvens*, *T. carboxydivorans*, *T. pseudospumae*, and *T. sunchonensis*, and performed “digital DDH” studies to determine their exact taxonomic positions using a phylogenomics approach.

## Materials and Methods

### Bacterial Strains

The six strains included in this study were type strains and obtained from four culture collections, with *T. pulmonis* CCUG 35732^T^ and *T. tyrosinosolvens* CCUG 38499^T^ obtained from Culture Collection, University of Gothenburg, Sweden (CCUG), *T. spongiae* DSM 44990^T^ from Leibniz Institute DSMZ—German Collection of Microorganisms and Cell Cultures, Germany (DSMZ), and *T. sunchonensis* JCM 15929^T^, *T. pseudospumae* JCM 13375^T^, and *T. carboxydivorans* JCM 15482^T^ from Japan Collection of Microorganisms (JCM), Japan.

### Phenotypic Characterizations

The six strains were phenotypically characterized in detail. Bacteria were streaked on Columbia agar with 5% defibrinated sheep blood and incubated at 25°C under aerobic condition for 2 days to observe the color of the colonies. Growth response to different temperatures (25, 37, and 42°C) were determined by spreading bacterial cells of 0.5 McFarland on Columbia agar with 5% defibrinated sheep blood agar followed by incubation under aerobic condition for up to 7 days. Hydrolysis of tyrosine and xanthine were tested as described previously (Conville and Witebsky, 2007). Biochemical data were obtained using the API 50 CH systems (bioMérieux, France) at 25°C according to manufacturers’ instructions. Reading intervals and durations of the tests followed the protocol suggested by Kattar et al. (2001). Cellular fatty acids were extracted directly from lyophilized cells (approximately 30 mg of original wet weight) grown on Columbia agar with 5% defibrinated sheep blood at 25°C for 2 days and analyzed by gas chromatography as described by Microbial ID, Inc. (Sasser, 1990) with peak-naming performed using MIDI, Inc. Sherlock Rapid Libraries and Methods.

### 16S rRNA Gene Sequencing

The six strains were subjected to 16S rRNA gene sequencing according to previously published protocol using primers LPW27807 (5′-TGGCTCAGGACGAACGCT-3′) and LPW27808 (5′-GAGGTGATCCAGCCGCA-3′; Teng et al., 2015). The sequences of PCR products were compared to known gene sequences in GenBank by multiple sequence alignment using Bioedit 7.1.9 (Hall, 1999). Phylogenetic tree was constructed by maximum likelihood method using the model GTR + I + G in MEGA 6.0 (Tamura et al., 2013).

### MALDI-TOF MS Analysis

MALDI-TOF MS of all *Tsukamurella* strains was performed as previously described (Verroken et al., 2010; Lau et al., 2014; Teng et al., 2014) with slight modifications. Briefly, bacteria were first grown aerobically on horse blood agar at 37°C for 2 days. Two to three colonies were scraped in 500 μl of distilled water and boiled for 30 min. After centrifugation at 13,000 rpm for 2 min, 300 μl of water followed by 900 μl of 100% ethanol was added and centrifuged at 13,000 rpm for 2 min twice. Supernatant was removed and pellet was air dried thoroughly. Fifty microliters of 70% formic acid was then added and mixed well with the pellet at room temperature for 15 min. Finally the solution was mixed with 50 μl of acetonitrile and centrifuged at 13,000 rpm for 2 min. One microliter of supernatant was loaded to a 96-well-spot polished steel target plate (Bruker Daltonik, Bremen, Germany) and air dried. One microliter of matrix solution (α-cyano-4-hydroxycinnamic acid; Sigma Aldrich, St Louis, USA) was applied and allowed to air dry. Within the range of *m/z* 2,000–20,000 Da, spectra were obtained with an accelerating voltage of 20 kV and collected in linear mode using the MicroFlex LT (Bruker Daltonik). Resulting data was analyzed with MALDI Biotyper 3.1 and a score of ≥1.7 and ≥2.0, as determined by the Reference Library V.3.1.2.0. Bruker Biotyper, was considered to be confident for identification on the genus and species level, respectively. Main spectral pattern (MSP) created from representative spectra of each strain were selected for hierarchical cluster analysis using the Bruker Biotyper software with default parameters (Ketterlinus et al., 2005).

### Wet-Lab DNA–DNA Hybridization

DDH studies were performed between *T. pulmonis* CCUG 35732^T^ and *T. spongiae* DSM 44990^T^, *T. tyrosinosolvens* CCUG 38499^T^ and *T. carboxydivorans* JCM 15482^T^ and *T. pseudospumae* JCM 13375^T^ and *T. sunchonensis* JCM 15929^T^, respectively. Preparation of genomic DNA was performed using QIAGEN Genomic tip 100-G (Qiagen, Hilden, Germany). DNA probe was prepared using reagent DIG-High Prime DNA labeling (Roche Diagnostics, Basel, Switzerland) according to Seong et al. (2003), while DNA–DNA dot blot hybridization was carried out using ULTRAhyb ultrasensitive hybridization buffer (Ambion, New York, USA), DIG wash and block buffers, anti-DIG-AP and CDP-Star (Roche Diagnostics) as described previously (Woo et al., 2014; Teng et al., 2015). DDH was performed at 42°C in triplicate for each of the probe used. To quantify the relative levels of DNA–DNA relatedness, developed X-ray films were scanned and dot intensities were analyzed using the software ImageJ 1.46r (Abràmoff et al., 2004). Self-hybridization values were considered to be 100%, representing the maximal achievable signal. Values obtained with other test strains were compared with this standard (Woo et al., 2014; Teng et al., 2015). Reciprocal hybridizations were also performed.

### Genome Sequencing, Assembly, and Annotation of the Six *Tsukamurella* Species

The draft genome sequences of the six *Tsukamurella* species were determined by high-throughput sequencing with Illumina Hi-Seq 2500. Genomic DNA of each strain was extracted from overnight cultures (37°C) grown on blood agars using the genomic DNA purification kit (Qiagen) as described previously (Woo et al., 2009; Tse et al., 2010). Extracted DNA was then sequenced by 151-bp paired-end reads with mean library size of 350 bp. Sequencing errors were corrected by k-mer frequency spectrum analysis using SOAPec^1^ *De novo* assembly was performed using SOAPdenovo2^2^ Prediction of protein coding regions and automatic functional annotation was performed using RAST (Rapid Annotations using Subsystem Technology) server version 2.0^3^ (Delcher et al., 2007; Aziz et al., 2008). Genomic islands were predicted by Zisland Explorer method^4^ (Wei and Guo, 2011; Wei et al., 2016).

### Phylogenomic Characterizations

The phylogenetic tree based on entire genome sequences was constructed by neighbor-joining method using GGDC distance (formula 2) and *Dietzia cinnamea* (NZ_AEKG01000298.1) as the root. The tree was saved in Newick format and subsequently processed by MEGA (Tamura et al., 2013). Intergenomic distance between these six *Tsukamurella* species was calculated using GGDC 2.1^5^ and ANI^6^ (Goris et al., 2007; Auch et al., 2010). The web service can be used for calculating intergenomic distances, with 70% (GGDC) and 94–96% (ANI) as the substitute for a wet-lab DDH value of 70% for same species delineation.

## Results and Discussion

### Phenotypic Characteristics

The biochemical and physiological properties of *T. pulmonis* CCUG 35732^T^, *T. spongiae* DSM 44990^T^, *T. tyrosinosolvens* CCUG 38499^T^, *T. carboxydivorans* JCM 15482^T^, *T. pseudospumae* JCM 13375^T^, and *T. sunchonensis* JCM 15929^T^ and are summarized in **Table 1.** Of the six *Tsukamurella* species, all were aerobic, non-sporulating, Gram-positive bacilli, and grew on sheep blood agar as white or orange to red colonies of <5 mm in diameter after 48 h of incubation at 25°C in ambient air. All of them grew well at 25 and 37°C but none of them grew at 42°C. *T. tyrosinosolvens* CCUG 38499^T^, *T. carboxydivorans* JCM 15482^T^, *T. pseudospumae* JCM 13375^T^, and *T. sunchonensis* JCM 15929^T^ could hydrolyse tyrosine but only *T. carboxydivorans* JCM 15482^T^ could hydrolyse xanthine. Sugar assimilation results using API 50 CH systems (bioMérieux, France) showed almost identical phenotypic profiles between *T. pulmonis* CCUG 35732^T^ and *T. spongiae* DSM 44990^T^ (48 of 49 sugar assimilation results were identical), between *T. tyrosinosolvens* CCUG 38499^T^ and *T. carboxydivorans* JCM 15482^T^ (48 of 49 sugar assimilation results were identical), and between *T. pseudospumae* JCM 13375^T^ and *T. sunchonensis* JCM 15929^T^ (45 of 49 sugar assimilation results were identical). The fatty acid profiles of the six *Tsukamurella* species are summarized in **Table 2.** The major fatty acids compositions of strains *T. pulmonis* CCUG 35732^T^, *T. spongiae* DSM 44990^T^, *T. tyrosinosolvens* CCUG 38499^T^, *T. carboxydivorans* JCM 15482^T^, *T. pseudospumae* JCM 13375^T^, and *T. sunchonensis* JCM 15929^T^ included C_16:0_, C_18:1ω9c_, and 10-methyl C_18:0_, which is typical of members of *Tsukamurella* (**Table 2**; Goodfellow and Kumar, 2012).

**Table 1:** Phenotypic properties of *T. pulmonis* CCUG 35732^T^, *T. spongiae* DSM 44990^T^, *T. tyrosinosolvens* CCUG 38499^T^, *T. carboxydivorans* JCM 15482^T^, *T. pseudospumae* JCM 13375^T^, and *T. sunchonensis* JCM 15929^T^.

Characteristics	*T. pulmonis* CCUG 35732^T^	*T. spongiae* DSM 44990^T^	*T. tyrosinosolvens* CCUG 38499^T^	*T. carboxydivorans* JCM 15482^T^	*T. pseudospumae* JCM 13375^T^	*T. sunchonensis* JCM 15929^T^
Colony color	White	Orange, red	White	White	Orange, red	Orange, red
**Hydrolysis of**						
Xanthine	–	–	–	+	–	–
Tyrosine	–	–	+	+	+	+
**Growth at**						
25°C	+	+	+	+	+	+
37°C	+	+	+	+	+	+
42°C	–	–	–	–	–	–
**Assimilation of ^∗^**						
Glycerol	+	+	+	+	+	+
D-Arabinose	+	+	+	+	–	–
D-Ribose	–	–	+	+	+	+
D-Galactose	+	+	+	+	+	+
D-Glucose	+	+	+	+	+	+
D-Fructose	+	+	+	+	+	+
D-Mannose	–	–	+	+	+	+
*myo*-Inositol	–	–	+	+	–	+
D-Mannitol	+	+	+	+	–	+
D-Sorbitol	+	+	+	+	–	+
*methyl*-D-Glucopyranoside	–	–	+	+	+	+
*N*-acetyl glucosamine	+	+	+	+	+	+
Arbutin	–	–	+	+	–	–
Esculin ferric citrate	+	+	+	+	+	+
D-Salicin	–	–	+	–	–	–
D-Cellobiose	–	+	–	–	–	–
D-Maltose	–	–	+	+	+	+
D-Saccharose	+	+	+	+	+	+
D-Trehalose	+	+	+	+	+	+
D-Melezitose	–	–	+	+	+	+
Xylitol	–	–	+	+	–	–
D-Turanose	+	+	+	+	+	+
L-Fucose	+	+	+	+	–	–
D-Arabitol	+	+	+	+	–	+
Potassium gluconate	+	+	+	+	+	+
2-Ketogluconate	–	–	+	+	–	+


*^∗^Negative results in all six isolates were not shown in this table. All results are obtained from this study.*

**Table 2:** Fatty acid compositions (%) of *T. pulmonis* CCUG 35732^T^, *T. spongiae* DSM 44990^T^, *T. tyrosinosolvens* CCUG 38499^T^, *T. carboxydivorans* JCM 15482^T^, *T. pseudospumae* JCM 13375^T^, and *T. sunchonensis* JCM 15929^T^.

	*T. pulmonis* CCUG 35732^T^	*T. spongiae* DSM 44990^T^	*T. tyrosinosolvens* CCUG 38499^T^	*T. carboxydivorans* JCM 15482^T^	*T. pseudospumae* JCM 13375^T^	*T. sunchonensis* JCM 15929^T^
C_14:0_	4.0	3.4	4.1	3.9	4.1	2.5
C_16:1ω9c_	2.5	1.1	2.6	2.0	2.4	1.6
C_16:0_	22.6	22.1	23.0	25.1	26.4	25.4
C_17:1ω8c_	–	–	1.2	1.2	–	–
C_17:0_	–	–	1.0	1.5	–	–
C_18:1ω9c_	34.9	36.4	32.8	33.5	36.3	27.4
C_18:0_	3.1	2.7	1.5	1.4	1.0	1.5
10-methyl C_18:0_	10.7	10.0	13.4	10.8	10.9	21.0
C_20:1ω9c_	3.3	1.1	1.8	2.0	1.6	1.2
SF1	15.2	19.5	16.4	15.2	15.6	16.2


*SF1 (summed feature 1): C16:1ω6c and/or C16:1ω7c and/or iso-C15:0 2-OH; All results were obtained from this study.*

*–, Less than 1% or not detected.*

### 16S rRNA Gene Sequencing

**FIGURE 1:**
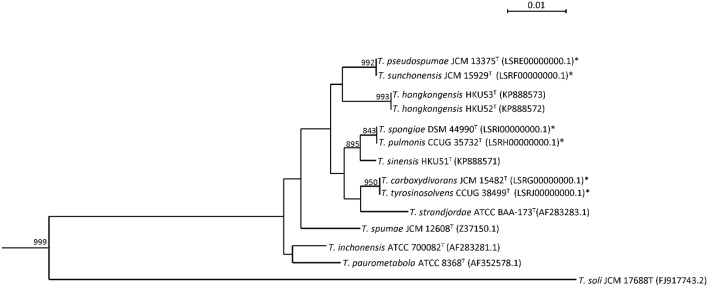
**Phylogenetic tree showing the relationship of *Tsukamurella* species using 16S rRNA gene sequence analysis.** The tree was constructed by maximum likelihood method using *D. cinnamea* (NZ_AEKG01000298.1) as the root. A total of 1407 nucleotide positions were included in the analysis. Bootstrap values were calculated from 1000 replicates. The scale bar indicates the number of substitutions per site. Names and accession numbers are given as cited in GenBank database. Sequences that were obtained in this study are marked by an asterisk.

Sequencing of the 16S rRNA gene of the six *Tsukamurella* species showed that there were 5–56 (0.3–3.8%) base differences between their 16S rRNA gene sequences and those of the type strains of other currently recognized *Tsukamurella* species. Pairwise sequence similarity calculations showed identical 16S rRNA gene sequences between *T. pulmonis* CCUG 35732^T^ and *T. spongiae* DSM 44990^T^, between *T. tyrosinosolvens* CCUG 38499^T^ and *T. carboxydivorans* JCM 15482^T^, and between *T. pseudospumae* JCM 13375^T^ and *T. sunchonensis* JCM 15929^T^ (**Figure 1**).

### MALDI-TOF MS Analysis

**FIGURE 2:**
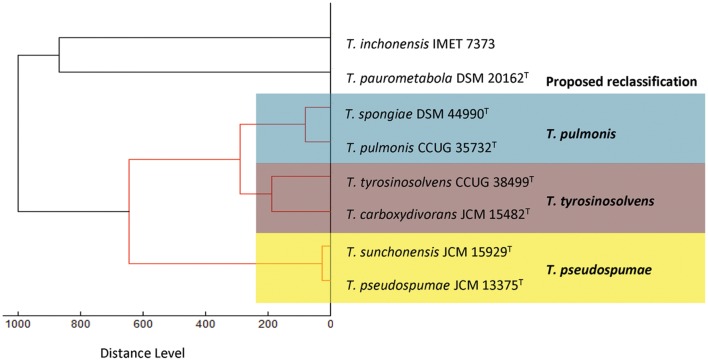
**Dendrogram generated from hierarchical cluster analysis of MALDI-TOF MS spectra of the six *Tsukamurella* species characterized in this study and the two *Tsukamurella* species included in the Bruker database.** Distances are displayed in relative units.

Consistent with the results of phenotypic characterization, dendrogram generated from hierarchical cluster analysis of MALDI-TOF MS showed that the spectra of these six *Tsukamurella* species and the representatives of the only two *Tsukamurella* species, *Tsukamurella inchonensis* and *Tsukamurella paurometabola*, available in the Bruker reference library revealed the same clustering pattern (**Figure 2**). Specifically, same cluster was observed between *T. pulmonis* CCUG 35732^T^ and *T. spongiae* DSM 44990^T^, between *T. tyrosinosolvens* CCUG 38499^T^ and *T. carboxydivorans* JCM 15482^T^, and between *T. pseudospumae* JCM 13375^T^ and *T. sunchonensis* JCM 15929^T^ (**Figure 2**).

### Wet-lab DNA–DNA Hybridization

Using the DDH, we found that DNA–DNA relatedness between *T. pulmonis* CCUG 35732^T^ and *T. spongiae* DSM 44990^T^, *T. tyrosinosolvens* CCUG 38499^T^ and *T. carboxydivorans* JCM 15482^T^, and *T. pseudospumae* JCM 13375^T^ and *T. sunchonensis* JCM 15929^T^ were demonstrated to be >70%, respectively. *T. pulmonis* CCUG 35732^T^ and *T. spongiae* DSM 44990^T^ possessed 91.1 ± 3.9% (mean ± SD, *n* = 3, *T. pulmonis* CCUG 35732^T^ as probe) or 84.2 ± 3.2% DNA–DNA relatedness (mean ± SD, *n* = 3, *T. spongiae* DSM 44990^T^ as probe). *T. tyrosinosolvens* CCUG 38499^T^ and *T. carboxydivorans* JCM 15482^T^ possessed 90.5 ± 1.3% (mean ± SD, *n* = 3, *T. tyrosinosolvens* CCUG 38499^T^ as probe) or 81.2 ± 0.6% DNA–DNA relatedness (mean ± SD, *n* = 3, *T. carboxydivorans* JCM 15482^T^ as probe). *T. pseudospumae* JCM 13375^T^ and *T. sunchonensis* JCM 15929^T^ possessed 95.1 ± 4.9% DNA–DNA relatedness (mean ± SD, *n* = 3, *T. pseudospumae* JCM 13375^T^ as probe) or 99.7 ± 1.0% (mean ± SD, *n* = 3, *T. sunchonensis* JCM 15929^T^ as probe). According to the criterion for species delineation using DDH, the present results (>70% DDH) suggested that these six *Tsukamurella* species should be reclassified as three distinct species. It is worth noting though, that the present DDH values are different from those obtained in the previous studies, in which the DNA–DNA relatedness between *T. pulmonis* CCUG 35732^T^ and *T. spongiae* DSM 44990^T^ was 48.0 ± 1.3% (mean ± SD, *n* = 3, *T. spongiae* DSM 44990^T^ as probe; Olson et al., 2007) and that of *T. tyrosinosolvens* CCUG 38499^T^ and *T. carboxydivorans* JCM 15482^T^ was 62.7 ± 3.4% (mean ± SD, *n* = 3, *T. carboxydivorans* JCM 15482^T^ as probe; Park et al., 2009). To further verify our results, we sequenced the genomes of these six *Tsukamurella* species for accurate species classification.

### General Features of the Genomes

**FIGURE 3:**
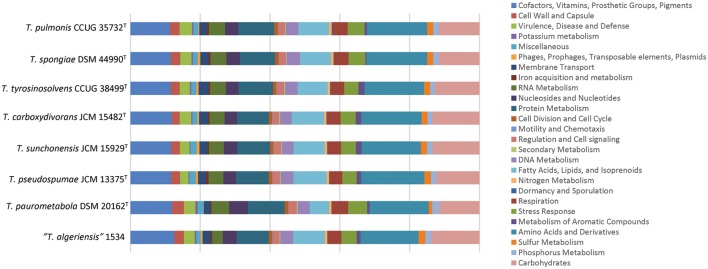
**Distributions of predicted coding sequence function in the annotated genomes according to RAST subsystems.** The draft genome sequences of the present six *Tsukamurella* species as well as those of *T. paurometabola* DSM 20162^T^ and “*T. algeriensis*” 1534 were analyzed. Each column indicates the number of CDSs of each *Tsukamurella* species in different subsystems showing in different color.

We sequenced the first draft genomes of the type strains of the six *Tsukamurella* species using high-throughput sequencing to investigate their genetic relatedness and to confirm their taxonomic positions. Sequencing generated 2.12 million paired-end reads per strain (estimated 80× coverage). After *de novo* assembly, the six draft genomes ranged from 4.6 to 5.2 Mb in length (G+C content 70.4–71.2%) distributed in 18–74 large contigs (>500 bp; GenBank accession numbers LSRE01000000–LSRJ01000000). All contigs generated for each strain were submitted to the RAST version 2.0 annotation server respectively, resulting in 4,412–5,191 protein-coding sequences (CDSs; **Table 3**). To facilitate the subsequent genomic analysis, genome sequences of *T. paurometabola* DSM 20162^T^ (GenBank accession number NC_014158.1) and “*T. algeriensis*” 1534 (GenBank accession number NZ_HE997626.1), the only two available genome sequences in GenBank, were also submitted to RAST for annotation. Each CDS in annotated genomes was grouped into different RAST subsystems based on the predicted functional role. Among the 4,412–5,191 CDS in these six draft genomes, only 1,629–1,762 CDSs can be categorized into RAST systems, representing 34–38% of total CDSs. Overall, the majority of CDSs were classified into subsystems of amino acid and derivatives (444–490 CDSs, 16.9–18.1%), carbohydrates (297–380 CDSs, 11.7–13.5%), cofactors, vitamins, prosthetic groups, pigments (294–335 CDSs, 11.4–11.9%) and protein metabolism (255–271 CDSs, 9.2–10.2%). The remaining 2,783–3,452 (62–66%) CDSs could not be classified into any subsystems, in which 1,450–1,720 (31.6–34.6%) CDSs were only annotated as hypothetical proteins. When we compared the distribution of CDSs in each subsystem of these six *Tsukamurella* genomes with those of *T. paurometabola* and “*T. algeriensis*” genomes, all of them have a similar percentage of their genome dedicated to each subsystem (**Figure 3**).

**Table 3:** Results of draft genome assembly of the six *Tsukamurella* species.

Genome assembly data	*T. pulmonis* CCUG 35732^T^	*T. spongiae* DSM 44990^T^	*T. tyrosinosolvens* CCUG 38499^T^	*T. carboxydivorans* JCM 15482^T^	*T. pseudospumae* JCM 13375^T^	*T. sunchonensis* JCM 15929^T^
Genome size (Mb)	4.74	4.60	5.23	4.95	4.95	5.21
G+C content (%)	70.4	71.1	71.2	71.2	70.6	70.5
No. of contigs	62	51	73	29	70	133
No. of predicted protein-coding genes	4,538	4,412	5,191	4,709	4,823	5,050


### Phylogenomic Analyses of the Six *Tsukamurella* Species

With the availability of these six *Tsukamurella* genome sequences, as well as the genome sequences of *T. paurometabola* DSM 20162^T^ and “*T. algeriensis*” 1534 from GenBank, we used two independent “digital DDH” methods, GGDC and ANI, to estimate the overall similarity between the genomes of two strains, which in turn determined the genome-based species delineation among these six *Tsukamurella* species. Consistent with the results obtained by traditional DDH, *in silico* genome-to-genome comparison unambiguously showed that for *T. pulmonis* CCUG 35732^T^ and *T. spongiae* DSM 44990^T^, *T. tyrosinosolvens* CCUG 38499^T^ and *T. carboxydivorans* JCM 15482^T^, and *T. pseudospumae* JCM 13375^T^ and *T. sunchonensis* JCM 15929^T^, GGDC and ANI values for more than 70 and 94–96%, respectively were observed (**Table 4**). As these calculated values are above the threshold for same species designation, it was suggested that the current six *Tsukamurella* species should be reclassified as only three distinct *Tsukamurella* species.

**Table 4:** Intergenomic distance between the draft genomes of the six currently recognized *Tsukamurella* species represented by ANI (upper triangle) and GGDC (lower triangle) values (%).

Strains	*T. pulmonis* CCUG 35732^T^	*T. spongiae* DSM 44990^T^	*T. tyrosinosolvens* CCUG 38499^T^	*T. carboxydivorans* JCM 15482^T^	*T. pseudospumae* JCM 13375^T^	*T. sunchonensis* JCM 15929^T^
*T. pulmonis* CCUG 35732^T^	–	**99.1**	87.3	87.2	84.6	84.8
*T. spongiae* DSM 44990^T^	**92.9**	–	87.3	87.3	84.5	84.8
*T. tyrosinosolvens* CCUG 38499^T^	34.3	34.3	–	**98.8**	85.1	85.1
*T. carboxydivorans* JCM 15482^T^	34.0	33.9	**90.4**	–	85.0	84.8
*T. pseudospumae* JCM 13375^T^	28.4	28.4	29.4	29.4	–	**98.1**
*T. sunchonensis* JCM 15929^T^	28.8	28.8	29.5	29.5	**82.3**	–


*Species with >70% GGDC and >94–96% ANI values, suggesting same species delineation, are bolded.*

**FIGURE 4:**
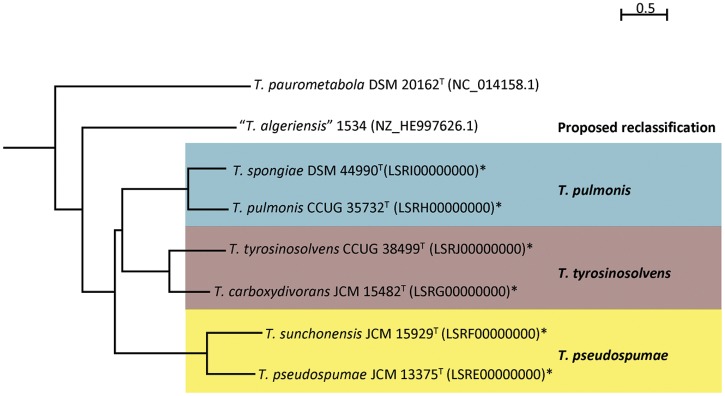
**Phylogenetic relationship among *Tsukamurella* strains with genome sequence available.** Neighbor-joining tree constructed on the basis of draft genome sequences GGDC distance (formula 2) and *D. cinnamea* (NZ_AEKG01000298.1) as the root. Scale bar indicates mean number of nucleotide substitutions per site on the respective branch. Names and accession numbers are given as cited in GenBank. Sequences that were obtained in this study are marked by an asterisk.

**FIGURE 5:**
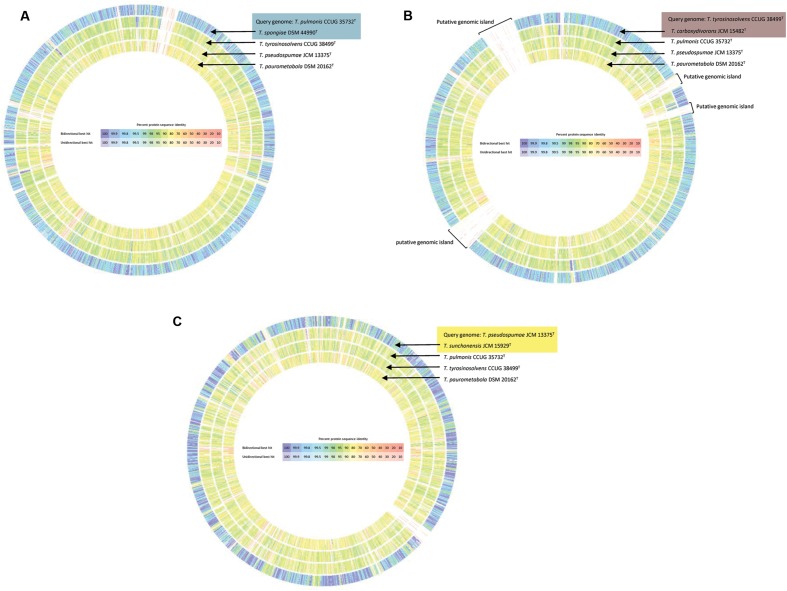
**Circular representation of the genome comparison between the draft genomes of the present study and that of *T. paurometabola* DSM 20162^T^ (NC_014158.1).** *T. pulmonis* CCUG 35732^T^ **(A)**, *T. tyrosinosolvens* CCUG 38499^T^ **(B)**, and *T. pseudospumae* JCM 13375^T^ **(C)** were used as query genome, respectively and compared with other selected bacterial genomes as indicated. Comparison was generated in RAST. Intensity of color indicates degree of protein identity, with blue to purple color indicating higher protein identity. *Tsukamurella* species with majority of orthologous genes sharing high protein identities to the query species was shaded using the same color.

The proposed reclassification is also supported by the phylogenomic analyses (**Figure 4**) and whole genome sequence comparison (**Figure 5**) using the draft genome sequences of the six *Tsukamurella* species. The phylogenetic tree constructed using the draft genomes sequences (**Figure 4**) was concordant to the clustering pattern as observed in phenotypic (**Table 1**) and MALDI-TOF MS analyses (**Figure 2**). As for genome sequence comparison, high protein sequence identities (>90% as indicated by blue color) were observed in each orthologous gene between the genomes of *T. pulmonis* CCUG 35732^T^ and *T. spongiae* DSM 44990^T^ (**Figure 5A**), between the genomes of *T. tyrosinosolvens* CCUG 38499^T^ and *T. carboxydivorans* JCM 15482^T^ (**Figure 5B**), and between the genomes of *T. pseudospumae* JCM 13375^T^ and *T. sunchonensis* JCM 15929^T^ (**Figure 5C**). Interestingly, although relatively high sequence similarity was observed between the genomes of *T. tyrosinosolvens* CCUG 38499^T^ and *T. carboxydivorans* JCM 15482^T^, some regions were found to be uniquely present in the type strain of *T. tyrosinosolvens* only. These unique regions are likely to be genomic islands as predicted by Zisland Explorer method (**Figure 5B**). RAST analysis showed that most of the ORFs within these putative genomic islands were annotated as hypothetic proteins, phage related proteins and mobile element proteins. As the type strain of *T. tyrosinosolvens* was isolated from blood culture of a patient with cardiac pacemaker implants, whereas *T. carboxydivorans* JCM 15482^T^ was isolated from soil, the pathogenic role of these genomic islands warrants further investigations. Given all genomic evidence, *T. spongiae* DSM 44990^T^, *T. carboxydivorans* JCM 15482^T^, and *T. sunchonensis* JCM 15929^T^ likely represent previously misclassified isolates that actually belong to the same species of *T. pulmonis*, *T. tyrosinosolvens*, and *T. sunchonensis*, respectively. According to Rules 23a and 42 of the Bacteriological Code (Lapage et al., 1992), we propose that *T. spongiae* Olson et al. 2007 (Olson et al., 2007), *T. carboxydivorans* Park et al. 2009 (Park et al., 2009), and *T. sunchonensis* Seong et al. 2008 (Seong et al., 2003) should be reclassified as later heterotypic synonyms of *T. pulmonis* Yassin et al. 1996 (Yassin et al., 1996), *T. tyrosinosolvens* Yassin et al. 1997 (Yassin et al., 1997), and *T. pseudospumae* Nam et al. 2004 (Nam et al., 2004), respectively.

## Proposal For A Reclassification of Species Within the Genus *Tsukamurella*

In this study, we sequenced the first draft genomes of six *Tsukamurella* species and unambiguously determined their phylogenetic positions using a phylogenomics approach. Consistent with the results of 16S rRNA gene sequencing, phenotypic and MALDI-TOF MS analyses, additional genomic evidences, including intergenomic distance comparisons using GGDC and ANI, phylogenetic analysis and whole genome comparison based on the draft genomes, support that the currently recognized six *Tsukamurella* species should be reclassified as three species only. According to Rules 23a and 42 of the *Bacteriological Code* (Lapage et al., 1992), it is proposed that *T. spongiae* Olson et al. 2007 (Olson et al., 2007) should be reclassified as a later heterotypic synonym of *T. pulmonis* Yassin et al. 1996 (Yassin et al., 1996), *T. carboxydivorans* Park et al. 2009 (Park et al., 2009) as a later heterotypic synonym of *T. tyrosinosolvens* Yassin et al. 1997 (Yassin et al., 1997), and *T. sunchonensis* Seong et al. 2008 (Seong et al., 2003) as a later heterotypic synonym of *T. pseudospumae* Nam et al. 2004 (Nam et al., 2004), respectively. With the decreasing costs of high-throughput sequencing genomic data may be considered as one of the taxonomic criteria for species delineation of bacteria, especially in the case where two strains share highly similar phenotypic characteristics and 16S rRNA gene sequences.

### Emended Description of *T. pulmonis* Yassin et al. 1996 (Yassin et al., 1996)

*Tsukamurella pulmonis* (pul. mo’ nis. L. gen. masc. n. pulmonis, of the lung, referring to the organ from which the bacterium was isolated).

#### Heterotypic Synonym: *T. spongiae* Olson et al. 2007 (Olson et al., 2007)

The characteristics of *T. pulmonis* are described by Yassin et al. (1996), with the following amendments. Grows best on Columbia agar with 5% defibrinated sheep blood agar as white or orange to red and rough or easily emulsified colonies, 2 mm in diameter, with irregular spreading margins after 48 h of incubation at 25°C under aerobic conditions. As determined by API 50 CH kit, cells are able to assimilate glycerol, D-arabinose, D-galactose, D-glucose, D-fructose, D-mannitol, D-sorbitol, *N*-acetyl glucosamine, esculin ferric citrate, D-saccharose, D-trehalose, D-turanose, L-fucose, D-arabitol, and potassium gluconate. The major fatty acids (>10% of the total) are C_16:0_, C_18:1ω9c_, 10-methyl C_18:0_ and summed feature 1 (C_16:1ω6c_ and/or C_16:1ω7c_ and/or iso-C_15:0_ 2-OH).

The type strain, CCUG 35732^T^ (=JCM 10111^T^ = DSM 44142^T^), was isolated from human with mycobacterial lung infection. The G+C content of the type strain is 70.4 mol%. The reference strain, DSM 44990 (=JCM 14882), is the nomenclatural type of *T. spongiae* Olson et al. 2007 (Olson et al., 2007), and was isolated from a deep-water marine sponge. The G+C content of strain DSM 44990 is 71.1 mol%. The GenBank accession numbers of whole genome sequence of the type strain and reference strain are LSRH00000000.1 and LSRI00000000.1, respectively.

### Emended Description of *T. tyrosinosolvens* Yassin et al. 1997 (Yassin et al., 1997)

*Tsukamurella tyrosinosolvens* (ty. ro. si. no. sol’ vens. Gr. masc. n. tyros, cheese; *tyrosine*, an amino acid; L. pres. part. *solvens*, dissolving; M.L. adj. *tyrosinoslovens*, tyrosine dissolving, referring to the hydrolysis of tyrosine which is characteristics of this species.

#### Heterotypic Synonym: *T. carboxydivorans* Park et al. 2009 (Park et al., 2009)

The characteristics of *T. tyrosinosolvens* are described by Yassin et al. (1997), with the following amendments. As determined by API 50 CH kit, cells are capable of assimilating glycerol, D-arabinose, D-ribose, D-galactose, D-glucose, D-fructose, D-mannose, *myo*-inositol, D-mannitol, D-sorbitol, *methyl*-D-glucopyranoside, *N*-acetyl glucosamine, arbutin, esculin ferric citrate, D-maltose, D-saccharose, D-trehalose, D-melezitose, xylitol, D-turanose, L-fucose, D-arabitol, potassium gluconate, and 2-ketogluconate. The major fatty acids (>10% of the total) are C_16:0_, C_18:1ω9c_, 10-methyl C_18:0_ and summed feature 1 (C_16:1ω6c_ and/or C_16:1ω7c_ and/or iso-C_15:0_ 2-OH).

The type strain, CCUG 38499^T^ (=JCM 10112^T^ = DSM 44234^T^), was isolated from blood culture of a patient with cardiac pacemaker implants. The G+C content of the type strain is 71.2 mol%. The reference stain, JCM 15482 (=KCCM 52885), is the nomenclatural type of *T. carboxydivorans* Park et al. 2009 (Park et al., 2009), and was isolated from soil sample from a roadside. The G+C content of strain JCM 15482 is 71.2 mol%. The GenBank accession numbers of whole genome sequence of the type strain and reference strain are LSRJ00000000.1 and LSRG00000000.1, respectively.

### Emended Description of *T. pseudospumae* Nam et al. 2004 (Nam et al., 2004)

*Tsukamurella pseudospumae* (pseu.do.spu’mae. Gr. adj. *pseudes* false; L. gen. n. *spumae* of foam and specific epithet of bacterial species; N. L. n. *pseudospumae* the false *spumae*, referring to the close relationship to *Tsukamurella spumae*).

#### Heterotypic Synonym: *T. sunchonensis* Seong et al. 2008 (Seong et al., 2003)

The characteristics of *T. pseudospumae* are described by Nam et al. (2004), with the following amendments. As determined by API 50 CH kit, cells are capable to grow on brain–heart infusion agar. Positive for hydrolysis of tyrosine. Assimilates glycerol, D-ribose, D-galactose, D-glucose, D-fructose, D-mannose, *methyl*-D-glucopyranoside, *N*-acetyl glucosamine, esculin ferric citrate, D-maltose, D-saccharose, D-trehalose, D-melezitose, D-turanose, and potassium gluconate. The major fatty acids (>10 % of the total) are C_16:0_, C_18:1ω9c_, 10-methyl C_18:0_ and summed feature 1 (C_16:1ω6c_ and/or C_16:1ω7c_ and/or iso-C_15:0_ 2-OH).

The type strain, JCM 13375^T^ (=DSM 44118^T^), was isolated from extensive foam in the aeration basin of an activated sludge process. The G+C content of the type strain is 70.6 mol%. The reference strain, JCM 15929 (=NRRL B-24668), is the nomenclatural type of *T. sunchonensis* Seong et al. 2008 (Seong et al., 2003), and was also isolated from activated sludge foam. The G+C content of strain JCM 15929 is 70.5 mol%. The GenBank accession numbers of whole genome sequence of the type strain and reference strain are LSRF00000000.1 and LSRE00000000.1, respectively.

## Data Accessibility

This Whole Genome Shotgun project has been deposited in DDBJ/ENA/GenBank under the accession numbers LSRE00000000–LSRJ00000000. The version described in this paper is version LSRE00000000.1–LSRJ00000000.1. The GenBank accession numbers for the draft genome sequences of *Tsukamurella pseudospumae* JCM 13375^T^, *Tsukamurella sunchonensis* JCM 15929^T^, *Tsukamurella carboxydivorans* JCM 15482^T^, *Tsukamurella pulmonis* CCUG 35732^T^, *Tsukamurella spongiae* DSM 44990^T^ and *Tsukamurella tyrosinosolvens* CCUG 38499^T^ are LSRE00000000.1, LSRF00000000.1, LSRG00000000.1, LSRH00000000.1, LSRI00000000.1, and LSRJ00000000.1, respectively.

## Author Contributions

JT conceived of the study, designed the study, carried out the molecular lab work, participated in data analysis, and drafted the manuscript; YT carried out the molecular lab work, participated in data analysis, and helped draft the manuscript. YH, F-BG, WW, and JC participated in data analysis; SW contributed reagents; SL revised the manuscript and contributed reagents; PW conceived of the study, designed the study, contributed reagents, and drafted the manuscript. All authors gave final approval for publication.

## Conflict of Interest Statement

The authors declare that the research was conducted in the absence of any commercial or financial relationships that could be construed as a potential conflict of interest.

## Footnotes

1 http://soap.genomics.org.cn/about.html.

2 http://soap.genomics.org.cn/.

3 http://rast.nmpdr.org/rast.cgi

4 http://cefg.uestc.edu.cn/Zisland_Explorer/

5 http://ggdc.dsmz.de/distcalc2.php

6 http://enve-omics.ce.gatech.edu/ani/
